# Neuropsychological Deficits in Disordered Screen Use Behaviours: A Systematic Review and Meta-analysis

**DOI:** 10.1007/s11065-023-09612-4

**Published:** 2023-09-11

**Authors:** Michoel L. Moshel, Wayne A. Warburton, Jennifer Batchelor, Joanne M. Bennett, Katherine Y. Ko

**Affiliations:** 1 School of Psychological Sciences, Macquarie University, Sydney, Australia; 2 School of Behavioural and Health Sciences, Australian Catholic University, Sydney, Australia

**Keywords:** Addiction gaming disorder, Internet gaming disorder, Internet addiction disorder, Attention and focus, Executive function, Cognitive testing

## Abstract

Over the last few decades, excessive and disordered screen use has become more prevalent, prompting investigations into its associated consequences. The extent to which disordered screen use behaviours impact neuropsychological functioning has been reportedly mixed and at times inconsistent. This review sought to synthesise the literature and estimate the magnitude of overall cognitive impairment across a wide range of disordered screen use behaviours. We also sought to determine the cognitive domains most impacted, and whether the observed impairments were moderated by the classification of screen-related behaviours (i.e., Internet or gaming) or the format of cognitive test administration (i.e., paper-and-pencil or computerised). A systematic search of databases (Embase, PsycINFO, MEDLINE) identified 43 cross-sectional articles that assessed neuropsychological performance in disordered screen use populations, 34 of which were included in the meta-analysis. A random-effects meta-analysis revealed significant small/medium (*g* = .38) cognitive deficits for individuals with disordered screen use behaviours relative to controls. The most affected cognitive domain with a significant medium effect size (*g* = .50) was attention and focus followed by a significant reduction in executive functioning (*g* = .31). The classification of disordered screen use behaviours into Internet or gaming categories or the format of cognitive testing did not moderate these deficits. Additionally, excluding disordered social media use in an exploratory analysis had little effect on the observed outcomes. This study highlights a number of methodological considerations that may have contributed to disparate findings and shows that disordered screen use can significantly impact cognitive performance. Recommendations for future research are also discussed. Data for this study can be found at https://osf.io/upeha/.

## Introduction

Technology and the Internet have provided innumerable benefits. However, excessive use without moderation may cause impairment in other areas of life. Despite current guidelines recommending no more than 2 h per day of recreational screen media for teenagers, including televisions, computers, and phones (Australian Institute of Health & Welfare, 2020), averages of more than 8 h per day have been recently reported (Cardoso-Leite et al., 2021). In excess, screen usage may exhibit many of the hallmark symptoms of other behavioural addiction disorders (Hwang et al., 2014; Warburton, 2021; Warburton et al., 2022) prompting debate and heterogeneity in the conceptualisation and classification of excessive and problematic screen use behaviours (Kuss et al., 2017; Marshall et al., 2022; Shaffer et al., 2000; Warburton & Tam, 2019). These disordered behaviours are sometimes described in terms of Internet addiction disorder (IAD; Li et al., 2018) or video gaming disorders (Ko et al., 2015; Warburton et al., 2022), but there is still disagreement about whether either classification accurately captures the scope of problematic behaviours (Király et al., 2015a). Nonetheless, there has been extensive research on the psychological, physical, and social consequences of screen-based disorders over the past few decades with findings highlighting detrimental impacts on health and overall wellbeing (Kircaburun et al., 2020; Kuss & Griffiths, 2012; Marshall et al., 2022; Paulus et al., 2018; Sugaya et al., 2019; Warburton, 2021; Warburton et al., 2022).

However, by contrast, there has been much less research and little consensus to date on the exact neuropsychological impacts that result from disordered screen use behaviours. Some studies report improvements in specific areas of cognition (Irak et al., 2016), whilst other studies report a reduction in those same areas (Cao et al., 2007). Inconsistencies in neuropsychological and neuroscientific methodologies have been identified as a potential contributor to such disparate findings (Pontes et al., 2017). The purpose of this review and analysis is to synthesise and quantify the effects of disordered screen use behaviours on neuropsychological outcomes, as well as explore the contribution of classification strategy and cognitive testing format on the measured outcomes.

### Operationalisation

Screen use is becoming increasingly recognised and investigated for its problematic aspects. For the purposes of this review, screen use refers to screen-based interactions including gaming (online and offline), Internet browsing, social media use, and smartphone use. In most cases, users interact with screens on a daily basis and engage with these technologies for work and leisure. However, some individuals may spend excessive amounts of time in front of a screen to the neglect and detriment of their social, physical, mental, and psychological wellbeing (Sigman, 2017). Some may even develop acute dependency symptoms similar to severe alcohol dependence (Hwang et al., 2014) or methamphetamine addiction (Jiang et al., 2020). Efforts have been made to characterise these problematic aspects of screen use in accordance with diagnostic classifications for other behavioural addictions such as gambling (Wölfling et al., 2020; Zhou et al., 2016). According to this characterisation, harmless screen use is seen to progress into the disordered and problematic realm when the following criteria are met: (1) screens are used excessively and with impaired control, (2) usage is associated with withdrawal when the screen is removed, (3) results in increased tolerance and the need to spend more time in front of a screen to satisfy the same desire, and (4) persists despite negative consequences to important areas of functioning such as increased social isolation, neglect in hygiene or health, progressive decline in other endeavours, or a downturn in academic or work performance (Sigman, 2017). Over the past decade, it has been observed that the prevalence of these symptoms has been increasing globally (Pan et al., 2020).

There has been much debate about how best to operationalise these addiction-like behaviours, with distinctions being made between the problem of screens as a whole and the problem of certain forms of screen use (Blaszczynski, 2006; Warburton, 2021). With regard to the latter, diagnostic classifications have been developed for specific screen-related usage such as social media addiction (Andreassen et al., 2016), technology addiction (Dadischeck, 2021), smartphone addiction (Yu & Sussman, 2020), Facebook addiction disorder (Brailovskaia et al., 2018), and various operationalisations of problematic Internet behaviours including Internet addiction (IA; Young, 2004), Internet disorder (Pontes & Griffiths, 2017), and problematic Internet use (PIU; Shapira et al., 2003). The only screen-based disorders to be officially classified are video game based: Internet gaming disorder (IGD), included in a section of the Diagnostic and Statistical Manual of Mental Disorders (DSM-5) for disorders requiring further study (American Psychiatric Association, 2013); gaming disorder (GD), included the 11th Revision of the International Classification of Disease (ICD-11; World Health Organization, 2019); and the sub-clinical hazardous gaming (HG), also in the ICD-11. The IGD and GD diagnoses both recognise excessive screen use as an addiction-like disorder rather than an issue of impulse control (e.g., see Pontes & Griffiths, 2014).

Although different types of maladaptive screen use can be considered as nosologically distinct in terms of impacted demographics (Pontes & Griffiths, 2014), it has been argued that all variants share the same basic diagnostic and etiological components characteristic of behavioural addictions (Griffiths, 2009a; Warburton, 2021; Weinstein, 2015). Of note, social media usage is generally considered distinct in terms of underlying motivations (Wolniewicz et al., 2018; Zhu & Xiong, 2022), affected cognitive domains (Weinstein, 2022), and aetiology (Pontes, 2017). However, with its recent emergence, research on its impacts and its potential similarity with other types of screen addiction is limited. At their core, the various diagnostic classification schemes ultimately refer to the numerous maladaptive and disordered activities associated with the use of screens. This is consistent with recommendations made by Pontes et al. (2017) to delineate between excessive screen time and “addicted” screen time, the latter characterised by functional impairment (also see Griffiths, 2009b). Moreover, from a clinical standpoint, the functional impairment resulting from disordered screen use, irrespective of the specific type of screen modality used, largely presents the same and is commonly treated in a comparable manner (Dell’Osso et al., 2021; Marshall et al., 2022; Warburton, 2021). Thus, it can be more helpful to conceptualise problematic screen use as on a continuum, with severe functional impairment at one extreme (Paulus et al., 2018; Warburton, 2021; Warburton et al., 2022). With the above in mind, this review will not limit its focus to specific diagnostic variants. Rather, it will consider disordered screen use behaviours in terms of broader categories of addiction-like behaviours marked by functional impairments.

### Functional Consequences

The psychological effects of disordered screen use behaviours have been extensively explored. For instance, screen-addicted individuals experience lower overall psychosocial wellbeing (Yang & Tung, 2007), increased psychiatric symptoms (Ha et al., 2006; Király et al., 2015b; Lai et al., 2015; Snodgrass et al., 2014; Vukosavljevic-Gvozden et al., 2015; Young & Rogers, 1998), lower life satisfaction (Samaha & Hawi, 2016), higher rates of loneliness (Yao & Zhong, 2014), compromised academic achievement (Hawi & Samaha, 2016; Jiang, 2014; Samaha & Hawi, 2016; Yang & Tung, 2007), reduced levels of sports and exercise (Henchoz et al., 2015), and poorer levels of health and sleep (Griffiths et al., 2004; Marshall et al., 2022; Wittek et al., 2016). In a large survey study involving around 15,000 teenagers, it was found that 5 or more hours of video gaming a day was significantly associated with higher reported instances of sadness, suicidal ideation, and suicidal plans compared to teenagers with no video game use (Messias et al., 2011). There is evidence that excessive screen time can cause a wide range of physical symptoms such as joint pain, strain injuries, peripheral neuropathy, encopresis, inflammation, and epileptic seizures (Chuang, 2006; Weinstein, 2010).

### Neuroimaging Research

There is also evidence that disordered screen use behaviours can impact neurostructural development (Schettler et al., 2022; Warburton, 2021). Engaging in excessive and obsessive video gaming during childhood can have significant structural and neuroadaptive impacts on reward-related, emotional-processing, and decision-making areas in the brain (Kuss & Griffiths, 2012; Schettler et al., 2022; Yao et al., 2017). Research has shown that individuals with gaming addictions have decreased grey and white matter volumes in areas associated with learning, reward, and memory proportional to their addiction duration, controlling for age, gender, and volume (Yuan et al., 2016, 2017). In a large population of children and young adults aged eight to 21, it was found that video game time was positively correlated with lower tissue density in cortical and subcortical areas observed over a 3-year period (Takeuchi et al., 2016). By comparing functional magnetic resonance imaging (fMRI) signals in response to cue-induced craving between gamers and non-gamers, Ko and colleagues (2009) found that the gamers exhibited stronger activation in the striatum and orbitofrontal cortex, regions commonly associated with other substance-related addictions. The same areas have been implicated in individuals diagnosed with Internet addiction (Dong et al., 2011). Temporal neuroimaging studies investigating the effects of disordered screen use behaviours, including the excessive and problematic use of social media and smartphones, demonstrate the emergence of atypical neural cue reactivity, aberrant activity (He et al., 2018; Horvath et al., 2020; Schmitgen et al., 2020; Seo et al., 2020), and altered neural synchronisation (Park et al., 2017; Youh et al., 2017). These features are seen to persist despite pharmacological treatment (Park et al., 2018). For a comprehensive review on neurobiological mechanisms and brain findings, see Weinstein et al. (2017) and Weinstein and Lejoyeux (2020); for impacts of excessive smartphone use, see Wacks and Weinstein (2021).

### Neuropsychological Consequences

Neuropsychological findings, designed to reflect neurobiological deficits, have not always mirrored the observed psychological or neurostructural and functional changes and have remained inconsistent. On the one hand, some studies have reported advantages: screen-addicted populations outperformed healthy controls on tasks assessing real-life decision-making, despite displaying higher novelty-seeking behaviours (Ko et al., 2010), made fewer errors and had quicker reactions on response inhibition tasks (Irak et al., 2016; Sun et al., 2009), and were superior at object recognition (Irak et al., 2016). In one study, it was found that even 10 h of video game experience was enough to improve performances on an attentional flexibility task in gaming naïve participants (Green & Bavelier, 2003). Other studies have found no difference in general intelligence (Hyun et al., 2015), risk-taking tendencies (Ko et al., 2010), or cognitive flexibility (Dong et al., 2010, 2014) in disordered screen use populations compared to healthy controls.

On the other hand, a number of studies reveal profound reductions within disordered screen use populations in many of the same areas of cognition. For one, several studies found decision-making to be markedly impaired in game-addicted populations including a propensity for immediate reward gratification and making disadvantageous and risky choices (Irvine et al., 2013; Pawlikowski & Brand, 2011; Tang et al., 2017; Wölfling et al., 2020; Yao et al., 2015). Cao and colleagues (2007) found that excessive Internet users showed greater impulsiveness as measured by self-rated scores and performed worse on a response inhibition task relative to controls. Attentional deficits have also been found with addicted gamers exhibiting a bias towards computer-related stimuli (e.g., laptop, computer keyboard, or mouse) characterised by an impaired disengagement of attention and protracted attentional processing (Heuer et al., 2021; Kim et al., 2018; Zhang et al., 2016; also see Kim et al., 2021). In fact, it has been found that individuals with disordered screen use behaviours share similar psychobiological mechanisms, neurocognitive impairments, and comorbidities with attention-deficit/hyperactivity disorder (ADHD), indicating a common neurofunctional deficit (Weinstein & Weizman, 2012; Weinstein et al., 2015; Yen et al., 2009). Indeed, there is a positive association between the amount of time children spend in front of screens daily and the severity of ADHD symptoms on a parent-rated scale (Chan & Rabinowitz, 2006). Time spent gaming was also found to be negatively correlated with overall cognitive performance, controlling for education and other demographics (Jang et al., 2021). These results stand in contrast to the above findings of enhanced cognitive performance or of no difference in discorded screen use populations.

Other reviews have questioned the heterogeneity in the literature regarding the impacts disordered screen use behaviours may have on cognition (Ko et al., 2015; Pontes et al., 2017). Firstly, when evaluating the neuropsychological impacts of disordered screen use behaviours, it is important to consider whether distinguishing between different diagnoses based on the predominant form of screen use is justified, or if the cognitive effects are largely uniform. That is, do different modalities of disordered screen use impact cognition differently? Does the interchangeability in defining and diagnosing disordered screen use behaviours obscure a reliable picture of cognitive outcomes? Second, Ko and colleagues (2015) pointed out that a good degree of cognitive functioning is a necessary requirement for performance on video games. The cognitive tasks that are used to assess impairment may draw on many of the same underlying cognitive capacities that are required for video gaming, and so may enhance performance rather than hinder it, potentially clouding conclusions where some authors report improvements and others report decrements. The authors caution against drawing premature conclusions about cognitive impacts based on studies that do not consider a broad range of cognitive tasks (also see Pontes et al., 2017). However, few studies implement a full battery of cognitive tasks, but instead infer domain-level impairments in “executive control” based on a single cognitive task (for example, see Wang et al., 2017). With this in mind, to determine cognitive outcomes as a result of disordered screen use, it is important to examine the role of disordered screen use classification with a focus on methodological issues in neuropsychological testing.

Further consideration should be given to the type and format of testing. The selection of tests is a crucial aspect of any assessment of cognition (Schoenberg & Scott, 2011; Strauss et al., 2006). Grounded in the literature, a test should be chosen based on its suitability for measuring a specific population under particular circumstances (Strauss et al., 2006). Depending on its psychometric properties, the type of test chosen can influence the accurate measurement of true impairment (Schoenberg & Scott, 2011). For instance, tests should be sensitive enough to capture the condition of interest but specific enough to avoid incorrectly classifying those who are unimpaired (Streiner, 2010). Although two tests may both measure executive functioning, only one of those tests may be sensitive enough to detect impairment in a given population. It is possible that whilst the Go/No-go task, for example, may fail to capture impairment in a disordered screen use population, the Stop Signal task may be better suited for this purpose.

Analysing cognitive performance in populations with disordered screen use also requires consideration of the test format: computerised or paper-and-pencil. Computerised administration is known to impact test performance, especially in individuals with high-computer anxiety and in some clinical populations (Browndyke et al., 2010; Strauss et al., 2006). In some cases, this may either mask true deficits (Strauss et al., 2006) or boost performances (Luciana, 2003). If individuals with disordered screen use behaviours demonstrate marked behavioural and neural attentional biases and disengagement from screen-related stimuli (see Heuer et al., 2021; Kim et al., 2018, 2021; Schmitgen et al., 2020; Zhang et al., 2016), one might reasonably expect differences in cognitive performance based on the format of testing. To the authors’ knowledge, whether the type or format of testing moderates cognitive performance has not been investigated to date.

### Aims

The inconsistencies apparent in the neuropsychological literature necessitate a quantitative examination of findings to illustrate the magnitude of cognitive deficit. This systematic review and meta-analysis will focus on neuropsychological considerations that may be contributing to the apparent discrepancies, such as number of cognitive tasks, type and format of testing, and assessment of disordered behaviours according to a predominant form of screen use (e.g., Internet or gaming). Previous reviews have focused on epidemiological research on screen addictions (Kuss et al., 2014; Pan et al., 2020), specific diagnostic variants without considering the similarities between disordered screen use behaviours (Legault et al., 2021), and deficits in narrow cognitive domains (Ioannidis et al., 2019; Yao et al., 2022), or only provided a qualitative analysis (Brand et al., 2014; Legault et al., 2021). Without holistic consideration of a comprehensive and inclusive integration of a wide range of screen technologies across multiple cognitive domains, this limits an accurate neuropsychological analysis of disordered screen use behaviours. With this aim in focus, our systematic review and meta-analysis seek to provide a comprehensive overview of cross-sectional studies examining neuropsychological comparisons between disordered screen-related behaviours and healthy controls, as well as to explore the quality of studies conducted up until now. In the meta-analysis, we also consider the contributions of disordered use classification (e.g., gaming, Internet, and social media), the type of tests, and the format of neuropsychological testing (e.g., computerised or manual).

## Methods

### Protocol and Registration

The systematic review was registered with PROSPERO on the 10th of December 2020 and amended the revision notes on the 15th of March 2022 to include plans for the meta-analysis, PROSPERO registration: CRD42020216147. The search was conducted following the PRISMA (Preferred Reporting Items for Systematic review and Meta-Analyses) guidelines (Page et al., 2021) and Gates and March’s (2016) recommendations for neuropsychological systematic reviews.

### Eligibility Criteria

The following criteria had to be met by studies to qualify for inclusion in the review: (1) the participants had to meet criteria or satisfy an operational definition for screen addiction, dependence, hazardous, excessive, or problematic screen use according to diagnostic measures or scales; (2) the disordered use group was compared to a group of healthy controls matched on a least one sociodemographic variable (age, gender, education); (3) at least one objective neuropsychological measure was used to assess cognitive functioning (e.g., not exclusively subjective self-reports or an analysis with an experimental manipulation); and (4) the study was available in English or translated into English. For studies to be included in the meta-analysis, they needed to provide sufficient data (i.e., means and SD, mean differences, Cohen’s *d*, Hedges’ *g* effect sizes, *t*-value, *p*-value).

Exclusion criteria were as follows: (1) results contained neuropsychological performance methods such as the Mini-Mental State Exam or the Barratt Impulsiveness Scale without an accompanying cognitive assessment; (2) either group had a comorbid diagnosis other than disordered screen use (e.g., ADHD or Autism Spectrum Disorder); (3) any single case studies; (4) exclusively neuroimaging studies without reporting on neuropsychological outcomes; (5) treatment or intervention studies with no cross-sectional data; (6) systematic reviews or meta-analyses; (7) grey literature including thesis abstracts, conference preliminary studies, or poster presentations; and (8) exclusively contained a non-screen-related diagnosis or operational definition (e.g., gambling). Studies were excluded from the meta-analysis if they (1) did not report (or respond to requests for) sufficient data to compute effect sizes; (2) contained assessment tasks that were modified or manipulated for experimental purposes such as only including addiction-related stimuli in a Stroop task and therefore tap into a different set of cognitive proc the type of tests, and the format of neuropsychological testing (see Brand et al., 2014); and (3) only included a test which was used once by that single study.

### Information Sources

A systematic literature search was conducted in December 2020 and additional studies were added until data extraction in November 2021. Searches were conducted independently in the following three databases: Embase, PsycINFO, and Medline.

### Search Strategy

The search strategy was developed and refined with the aid of an experienced librarian. Like Paulus et al. (2018), we conceptualised disordered screen use behaviour broadly in order to maximally capture the various and inconsistent definitions throughout the literature. We placed no restrictions on language or publication date. A restriction was placed on human studies. A combination of the following keywords was used: (“internet*” or “online” or “web” or “computer” or “screen*” or “mobile phon*” or “smartphon*” or “gaming” or “games” or “video gam*” or “television” or “tv” or “social media”) and (“addict*” or “dependen*” or “excess*” or “problematic*” or” disorder*” or “hazardous*” or “obsess*” or “overus*” or “impair*”) and (“neuropsyc*” or “memory” or “attentt*” or “intelligen*” or “cognit*” or “executive function*”).

### Selection Process

Two authors (MM and KK) independently reviewed the relevant articles at each distinct stage of identification, screening, eligibility, and inclusion. Reference lists of relevant studies were examined, and studies included if they met the relevant criteria. Disagreement about inclusions between the two reviewers was resolved through discussion and, if unresolved, was examined by a third author (JB or WW).

### Data Collection Process

Data were extracted into Microsoft Excel and independently cross-checked by two authors (MM and KK). For studies that reported more than one comparison group (e.g., healthy control and ADHD), only the healthy control group was used as a comparison (Wollman et al., 2019). Additionally, in the instances where cognition was assessed more than once (e.g., longitudinal or intervention studies), only the baseline cross-sectional data were extracted. Nine authors were contacted to clarify either methodology or relevant criteria, or to request data required to compute effect sizes. Two authors (Metcalf & Pammer, 2014; Park et al., 2011) responded with the required data and were included in the systematic review and meta-analysis. Among the seven remaining studies, two were excluded from the systematic review because of insufficient information regarding eligibility requirements, and the rest were included only in the systematic review but not the meta-analysis.

### Data Items

Variables extracted included the (1) year of publication, (2) country of publication, (3) demographic information (sample size, mean and standard deviation of education and age, and number of males and females in the sample when available), (4) disordered behaviour classification (e.g., IGD, IAD, or PIU), (5) associated measure including cut-offs when available, (6) assessment of cognitive performance, and (7) format of cognitive assessment (e.g., computerised or manual). For data only reported in figures, we extracted the relevant values using WebPlotDigitizer (Rohatgi, 2021) to ensure maximal inclusion (Pick et al., 2019). For studies that did not report means or standard deviations, we extracted either *t*-values, *p*-values, or effect sizes.

To examine neuropsychological domains separately for the meta-analysis, cognitive tests were grouped into the domains of global functioning, executive functioning, processing speed, attention, and working memory according to clinical guidelines (Strauss et al., 2006) and previous reviews (Mauger et al., 2018; Shin et al., 2014; Wagner et al., 2015). However, it is acknowledged that many tests are not pure measures of any given cognitive domain but share underlying similarities and are only, therefore, imperfect indicators of cognitive ability within domains (Engle et al., 1999; Rabaglia et al., 2011). The domain of executive functioning was used broadly to refer to the abilities involved in problem-solving, goal-directed behaviours, inhibitory control, cognitive flexibility, planning, concept formation, and strategy generation (Elliott, 2003; Miyake et al., 2000). Tasks that required psychomotor, visuomotor, or decision speed abilities were grouped under the processing speed domain (Strauss et al., 2006). Tests that assessed rapid response selection, attentional capacity, and sustained performance were grouped under the attention domain (Strauss et al., 2006). Finally, tasks that required retaining and manipulation of information over the short term were grouped under the working memory domain (Strauss et al., 2006). In the cases where a single test produced more than one outcome (e.g., Digit Span or Go/No-go), the outcomes were sorted into their relevant domain (e.g., Digit Span forwards under attention and Digit Span backwards under working memory). Cognitive tests that were used only once (e.g., the Cups Task) were unsuitable for a meta-analysis and were not included. This limited the number of possible cognitive domains for inclusion in the analysis such as memory, language, and visuospatial skills.

### Study Risk of Bias Assessment

For quality assessment, we used the National Heart, Lung, and Blood Institute (NHLBI) Quality Assessment Tool for Observational Cohort and Cross-Sectional Studies (NHLBI, 2019). In the current analysis of cross-sectional data at a single time point, only eight out of fourteen methodological criteria from the assessment tool were applicable. In accordance with Carbia et al. (2018), we adapted Item 5 to better capture the quality of the sample size (*n* ≥ 25), known to be important when sample size calculations were not computed (Grjibovski et al., 2015; Wang & Cheng, 2020). Two independent authors (MM and KK) evaluated each of the items with “yes”, “no”, “cannot determine”, “not reported”, or “not applicable”. After independent evaluation of each study, disagreements were resolved through discussion until a consensus was reached. Given the modification of the scale for the purposes of this review and consistent with recommendations, we did not include an overall rating summary (O’Connor et al., 2015; Robinson et al., 2021; Sanderson et al., 2007).

### Effect Measures

The effect size of standardised mean differences in cognitive performance between the controls and the disordered use group was calculated and expressed as Hedges’ *g* and its 95% confidence interval (95% CI) (Hedges, 1981; Hedges & Olkin, 1985). Hedges’ *g*, a variation of Cohen’s *d*, was used to correct for potential bias related to the sample sizes in individual studies and the resultant overestimation of true population effects (Hedges & Olkin, 1985). As with Cohen’s *d*, a Hedges’ *g* effect size of 0.20 represents a small effect, 0.50 a medium effect, and 0.80 a large effect (Cohen, 1988). Higher Hedges’ *g* scores indicated a greater difference between the disordered use group and the control group reflecting an inferior performance of the former.

### Synthesis Methods

The meta-analysis was conducted using the Comprehensive Meta-Analysis (CMA) version 3 software package (Borenstein et al., 2013). In line with Borenstein et al. (2013), we selected a random-effects model given that significant heterogeneity of effects was expected beyond sampling error and the included studies varied with respect to sample characteristics and cognitive tasks. All analyses were examined for heterogeneity by using Tau-squared, *I*-squared, and *Q*-squared statistics. Consistent with Higgins et al. (2003), we interpreted an *I*^2^ of 25% as low, 50% as moderate, and 75% as high heterogeneity. Based on recommendations by Borenstein et al. (2021) and to ensure that there was sufficient power for moderator variables, studies had to have (1) at least two of the same cognitive tasks or outcome measures and (2) at least two of the same disorder classification groups to be included in subgroup analyses.

### Reporting Bias Assessment

In assessing the risk of bias between studies, four methods were applied that assessed the overall effect and all subgroup analyses. To quantify asymmetry and identify small-study effects, funnel plots were visually inspected for symmetry around the combined effect size, and Egger’s test of the intercept was computed. This was supported by Duval and Tweedie’s trim and fill analysis which provides an estimate of the number of missing studies and adjusts the estimated overall effect size (Duval & Tweedie, 2000). Finally, classic fail-safe N was used to calculate the minimum number of undetected negative results that were necessary to nullify the effect (e.g., to raise the observed *p*-value above 0.05).

## Results

### Study Selection

**Fig. 1:**
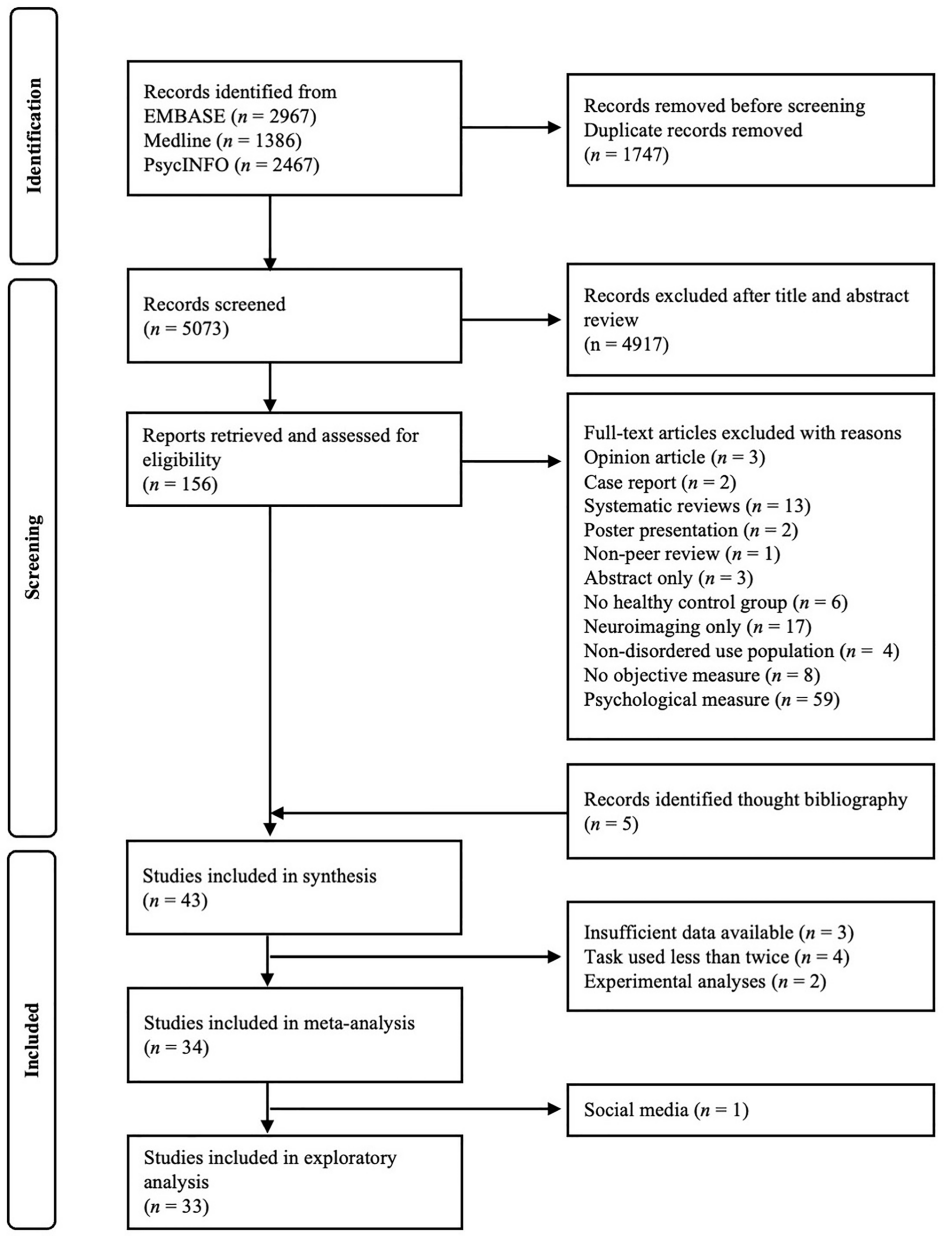
A PRISMA flow diagram illustrating the search strategy, study screening, selection, exclusion, and inclusion of studies in the systematic review and meta-analysis

Figure 1 presents a flow chart illustrating the identification, screening, and final inclusion of all studies in the review and analysis process. Five additional studies identified through reference list searches were added based on inclusion criteria. A total of 43 studies satisfied the eligibility criteria for inclusion in the systematic review and 34 studies were included in the meta-analysis. Of those, 33 were included in an exploratory analysis.

### Study Characteristics

The extracted summary data from included studies are shown in Table 1. Summary data included country, participant, and control descriptive data (age, sex, and education); disordered use classification and measure; neuropsychological assessment; and format of testing. Almost half of the included studies were conducted in China (*n* = 20). Eight studies were from Europe (Germany (*n* = 5), Spain (*n* = 1), Netherlands (*n* = 1), and Bosnia and Herzegovina (*n* = 1); six studies from South Korea; two from Taiwan; two from Turkey; two from the UK; one from Iran; one from Australia; and one from Brazil. The majority of studies were conducted between 2014 and 2018 consistent with IGD’s first appearance in the DSM-5 in May 2013. The included studies yielded a total of 1341 participants with screen disorders (72% males) and 1590 healthy controls (69% males). The results of some studies were not reported separately for disordered use and control groups, so the demographic of the entire group was included.

**Table 1:** Study characteristics

**Study**	**Country**	**Participant, *N***	**Control, ***N*	**Behaviour classification**	**Behaviour measure**	**Cognitive measure**	**Format**
		**Age, *M* (*SD*)**	**Age, *M* (*SD*),**				
		**Education *M* (*SD*)**	**Education *M* (*SD*)**				
		**Sex (%m)**	**Sex (%m)**				
Aydın et al. (2020)^a, b^	Bosnia and Herzegovina (Turkish students)	100	140	PSNSU	BSMAS (19/30)	WCST	Computerised
		22.16 (3.50)	23.42 (5.19)				
		14.53 (2.06)	15.35 (1.90)				
		50.0%	50.7%				
Cai et al. (2016)^a^	China	27	27	IGD	DSM-5 (≥ 5) IGD, IAT (> 50)	Stroop	Computerised
		17.9 (0.9)	18.3 (1.6)				
		12.0 (0.8)	12.4 (1.4)				
		85.2%	73.3%				
Choi et al. (2013)^a^	South Korea	21	20	IAD	IAT (> 70), > 4 h/day	SST	Computerised
		23.33 (3.50)	22.40 (2.33)				
		14.81 (1.94)	14.95 (1.36)				
		57.1%	55.0%				
Choi et al. (2014)^a^	South Korea	23	24	IAD	IAT (> 70), 4 h/day and 30 h/week	K-WAIS-SF, LNST, Stroop, TMT, VF, IED, SOC, SSP, SST	Computerised and manual
		23.22 (3.44)	22.42 (2.47)				
		14.68 (1.91)	15.00 (1.47)				
		52.2%	54.2%				
Collins and Freeman (2014)	UK	20	20	PVGP	GAS (≥ 4)	Enumeration paradigm, mental rotation task, task switching paradigm, VSTM, flanker compatibility task	Computerised
		23.85 (6.10)	21.93 (3.15)				
		NR	NR				
		90.0%	50.0%				
Ding et al. (2014)^a^	China	17	17	IGA	DSM-4, YDQ (Q1-5 and ≥ 1 on remainder)	GNG	Computerised
		16.41 (3.20)	16.29 (2.95)				
		8.82 (3.09)	9.53 (3.43)				
		82.5%	82.5%				
Dong et al. (2010)^a^	China	12	12	IAD	IAT (≥ 7)	GNG	Computerised
		20.47 (4.12)	20.19 (4.47)				
		NA	NA				
		100%	100%				
Dong et al. (2014)^a^	China	15	15	IAD	IAT (≥ 80)	Stroop	Computerised
		21.6 (3.0)	22.4 (3.3)				
		NA	NA				
		100%	100%				
Dong et al. (2015)	China	35	36	IGD	IAT (≥ 50)	Stroop	Computerised
		22.21 (3.08)	22.81 (2.36)				
		NA	NA				
		100%	100%				
Dong et al. (2017)^a^	China	18	19	IGD	DSM-5, IAT (≥ 50)	Stroop, guessing task	Computerised
		21 (2.83)	21 (3.67)				
		NA	NA				
		100%	100%				
Han et al. (2012)^a^	South Korea	20	18	POGA	YIAS (> 50), > 4 h/day and 30 h/week	WCST	Computerised
		20.9 (2.0)	20.9 (2.1)				
		12.0 (1.7)	12.1 (1.1)				
		100%	100%				
Irak et al. (2016)^a^	Turkey	41	32	IGD	GAS (> 55), PGASL (> 3), > 16 h/week	Working memory task, object recognition task, VSMT, GNG, emotional memory task	Computerised
		21.9 (3.02)	23.9 (2.57)				
		85.7% undergrad, 14.3% postgrad					
		55.1%					
Irvine et al. (2013)^a^	England	26	26	PVG	GAS, YBOCS adaption, DSM-IV adaption	IST, DDT, SST, PRT, NART	Computerised
		24.69 (5.90)	25.61 (5.87)				
		NR	NR				
		88.5%	88.5%				
Jang et al. (2021)^a^	Korea	64	63	IGD	DSM-5, IAT	WAIS-IV	Manual
		24.45 (5.44)	24.27 (3.16)				
		13.23 (1.48)	14.35 (1.81)				
		90.6%	74.6%				
Jeromin et al. (2016a)	Germany	21	30	EIG	CIUSWoW (> 25)	Addiction Stroop, visual probe	Computerised
		22.9 (2.1)	24.5 (3.2)				
		NA	NA				
		81%	63.3%				
Jeromin et al. (2016b)	Germany	27 and 29	27 and 29	IGD	IGDQ (> 5)	Addiction Stroop	Computerised
		24.9 (7.4) and 31.2 (7.7)	23.3 (5.3) and 23.5 (4.9)				
		NA	NA				
		70.4% and 100%	70.4% and 100%				
Khoury et al. (2019)^a^	Brazil	47	43	SA	SPAI-BR	IGT, GDT, Raven’s progressive matrices	Computerised
		22.39 (± 1.67)					
		NA	NA				
		46.8%	48.8%				
Kuo et al. (2018)^a^	Taiwan	35	39	IA	CIAS (> 15%ile)	Stroop, WCST, WDST	Manual
		11.6 (0.5)	11.3 (0.7)				
		5.5 (0.5)	5.3 (0.5)				
		77.1%	20.5%				
Li et al. (2020)^a^	China	31	32	IGD	YDQ (Q1-5 and ≥ 1 on remainder)	GNG, gambling task	Computerised
		15.81 (1.68)	15.91 (1.73)				
		9.42 (1.71)	10.00 (1.83)				
		90.3%	90.6%				
Lim et al. (2016)^a^	South Korea	44	40	IGD	DSM-5, IAT (≥ 70), 4 h/day and 30 h/week	K-WAIS-SF, IED, SOC, SSP, SST, Stroop, TMT, VF	Computerised and manual
		19.16 (5.22)	21.37 (6.31)				
		11.25 (2.48)	12.13 (3.32)				
		100%	100%				
Liu et al. (2014)^a^	Taiwan	11	11	IGD	DCIA-C	GNG	Computerised
		23.45 (2.34)	23.45 (2.34)				
		16.09 (1.22)	16.18 (1.40)				
		100%	100%				
Luijten et al. (2015)^a^	Netherlands	18	16	PG	VGAT (≥ 2.5)	GNG, Stroop	Computerised
		20.83 (3.05)	21.38 (3.03)				
		Low: 61% Med: 33% High: 6%	Low: 50% Med: 44% High: 6%				
		100%	100%				
Marin Vila et al. (2018)^a^	Spain	113	462	PIU	IREQ (≥ 34)	WAIS-III SS, SDMT, VF, DAT, immediate/delayed recall task	Manual
		14.79 (0.86)	14.63 (0.78)				
		51.8% 3rd year of high school	56.6% 3rd year of high school				
		59.3%	57.6%				
Metcalf et al. (2014)a	Australia	22	22	AFPSG	AEQ (≥ 4)	GNG, IGT, CPT	Computerised
		22.81(4.03)	24.86 (5.78)				
		NA	NA				
		100%	100%				
Müller et al. (2021)	Germany and Spain	56	50	PSNSU	sIAT for SNS (≥ 35)	MCST, CLT	Computerised
		20.63(1.95)	22.70(3.25)				
		NA	NA				
		21%	36%				
Park et al. (2020)^a^	South Korea	34	34	IGD	DSM-5, IAT, ≥ 4 h/day and ≥ 30 h/week	WAIS- IV, GNG, CGT, SST, Stroop	Computerised
		25.5 (4.3)	25.9 (6.0)				
		14.7 (1.9)	14.2 (1.9)				
		88.2%	88.2%				
Pawlikowski et al. (2011)^a^	Germany	19	19	EIG	IATwow (> = 50)	GDT	Computerised
		23.47(3.88)	24.32(3.62)				
		13	13				
		73.7%	73.7%				
Shafiee-Kandjani et al. (2020)^a^	Iran	27	26	IAD	YIAT, DSM-IV	SDMT, LNST, TMT, VF, Stroop, DST	Manual
		27.70 (7.22)	30.62 (6.27)				
		38.46% Bachelors	26.92% Bachelors				
		51.8%	61.5%				
Sun et al. (2009)^a^	China	52	61	EIU	IAT (EIU ≥ 5)	Gambling task, GNG	Computerised
		21.5 (2.3)	20.7 (2.1)				
		NR	NR				
		80.8%	81.9%				
Tekin et al. (2018)^a^	Turkey	30	29	IA	IAS (≥ 81)	Stroop, TMT	Manual
		20.92 (2.78)					
		NR	NR				
		40%	41.4%				
Wang et al. (2015)^a^	China	28	28	IGD	YDQ (Q1-5 and ≥ 1 on remainder)	Stroop	Computerised
		18.8 (1.33)	19.3 (2.56)				
		12.18 (0.48)	12.2 (0.56)				
		64.3%	71.4%				
Wang et al. (2017)^a^	China	18	21	IGD	IAT (≥ 50)	DDT	Computerised
		23.1 (2.0)	22.1 (3.2)				
		NA	NA				
		100%	100%				
Wang et al. (2020)	China	24	23	IAD	IAT (≥ 50), 10 h/day and 6 days/week	2-back task	Computerised
		20.58 (2.39)	21.13 (2.51)				
		NA	NA				
		58.3%	43.5%				
Wölfling et al. (2020)^a^	Germany	30	27	IGD	Lie/Bet-Questionnaire (≥ 1), BIG-S (≥ 5), AICA-C gaming (≥ 7)	DDT, IGT	Computerised
		26.9 (5.97)	25.6 (3.25)				
		60.0% higher education	92.6% higher education				
		100%	100%				
Wu et al. (2020)	China	26	28	IGD	DSM-5 (≥ 5), ≥ 20 h/week for at least 6 months	Stroop	Computerised
		21.77 (2.61)	20.96 (1.95)				
		16.35 (2.23)	15.46 (1.45)				
		53.8%	46.4%				
Xing et al. (2014)^a^	China	17	17	IGD	IAT (> 50)	Stroop	Computerised
		19.1 (0.7)	19.8 (1.3)				
		12.2 (0.6)	12.4 (0.7)				
		58.8%	64.7%				
Yao et al. (2015)	China	60	42	IGD	CIAS (≥ 67), ≥ 14 h/week for at least 1 year	The Cups Task	Computerised
		22.40 (2.07)	22.38 (2.10)				
		15.60 (1.81)	15.60 (1.85)				
		100%	100%				
Yuan et al. (2016)^a^	China	20	20	IGD	YDQ	Stroop	Computerised
		NR for behavioural					
Yuan et al. (2017)^a^	China	43	44	IGD	DSM-5 (≥ 5)	Stroop	Computerised
		19.0 (1.4)	19.5 (1.8)				
		11.5 (0.6)	11.4 (0.7)74.4%				
		74.4%	77.2%				
Zhou et al. (2010)^a^	China	26	26	PIU	YDQ (Q1-5 and ≥ 1 on remainder)	GNG	Computerised
		25 (6)	25 (6)				
		NR	NR				
		73.0%	73.0%				
Zhou et al. (2013)	China	23	23	IAD	YDQ (Q1-5 and ≥ 1 on remainder)	Modified Eriksen flanker task	Computerised
		25 (6)	25 (6)				
		9 (4)	9 (4)				
		73.9%	73.9%				
Zhou et al. (2014)^a^	China	22	22	IAD	YDQ (Q1-5 and ≥ 1 on remainder)	GNG, WCST, DST	Computerised
		28 (7)	28 (7)				
		9(3)	9(3)				
		72.7%	68/2%				
Zhou et al. (2016)^a^	China	23	23	IAD	YDQ (Q1-5 and ≥ 1 on remainder)	DST, WCST, GNG	Computerised
		29 (7)	28 (6)				
		9(3)	9(3)				
		73.9%	69.5%				

*AEQ* Addiction-Engagement Questionnaire, *AFPSG* Addicted First-Person Shooter Gaming, *AICA-C* checklist for the Assessment of Internet and Computer game Addiction, *BIG-S* The Berlin Inventory of Gambling behavior–Screening, *BSMAS* Bergen Social Media Addiction Scale, *BSAI* Brazilian Smartphone Addiction Inventory, *CGT* Cambridge Gambling Task, *CIAS* Chinese Internet Addiction Scale, *CIUSWoW* Compulsive Internet Use Scale for WoW, *CPT* continuous performance task, *CLT* Cards and Lottery Task, *DAT* Differential Aptitude Test, *DCIA-C* Diagnostic Criteria for Internet Addiction for College Students, *DDT* Delay Discounting task, *DSM* Diagnostic and Statistical Manual of Mental Disorders, *DST* Digit Span test, *EIG* excessive Internet gaming, *EIU* excessive Internet use, *GAS* Game Addiction Scale, *GDT* Game of Dice task, *GNG* Go/No‐go Task, *IA* Internet addiction, *IAD* Internet addiction disorder, *IAS* Internet Addiction Scale, *IAT* Young’s Internet Addiction Test, *IED* Intra-Extra Dimensional Set Shift, *IGA* Internet gaming addiction, *IGD* Internet gaming disorder, *IGDQ* Internet Gaming Disorder Questionnaire, *IGT* Iowa Gambling Task, *IREQ* Internet-Related Experiences Questionnaire, *IST* Information Sampling Task, *LSNT* Letter–Number Sequencing, *MCST* Modified Card Sorting Test, *NA* not assessed, *NART* National Adult Reading Test, *NR* not reported, *PIU* problematic Internet use, *PG* problematic gamers, *PGASL* Pathological Game Addiction Symptoms List, *POGA* patients with on-line game addiction, *PRT* premature responding task. *PSNSU* Problematic Social Networking Sites Use, *PVG* pathological video game use, *PVGP* problematic video game playing, *SA* smartphone addiction, *SDMT* Symbol Digit Modalities Test, *sIAT-SNS* Short Internet Addiction Test specified for social networking sites, *SOC* Stockings of Cambridge test, *SPAI-BR* Brazilian Smartphone Addiction Inventory, *SS* Symbol Search Subtest, *SSP* Spatial Span test, *SST* Stop Signal Task, *TMT* Trail Making Test, *VF* verbal fluency, *VGAT* Videogame Addiction Test, *VSMT* Visual spatial memory task, *WAIS-SF* Short form of Korean-Wechsler Adult Intelligence Scale, *WCST* Wisconsin Card Sorting Task, *WDST* Wechsler Digit Span test, *WOW* World of Warcraft, *YBOCS* Yale-Brown Obsessive–Compulsive Scale, *YDQ* modified Young Diagnostic Questionnaire for Internet addiction, *YIAS* Young’s Internet addiction scale

^a^Included in the meta-analysis

^b^excluded in exploratory analysis

Sample sizes differed considerably between studies, with the smallest study incorporating 11 participants (Liu et al., 2014) and the largest involving 113 participants (Marín Vila et al., 2018). There was less variability in age between studies with the youngest average age included being 11 years (Kuo et al., 2018) and the oldest 29 years (Zhou et al., 2016). As with Casale et al. (2021), this review grouped different age samples together given the similarity of technology-related problems across ages. There was a disproportionate number of males with some studies including only males (Dong et al., 2010, 2014, 2015, 2017; Han et al., 2012; Jeromin et al., 2016a, 2016b; Lim et al., 2016; Liu et al., 2014; Luijten et al., 2015; Metcalf & Pammer, 2014; Wang et al., 2017; Wölfling et al., 2020; Yao et al., 2015). Years of education between studies ranged from 5.6 (Kuo et al., 2018) to 21.5 years (Dong et al., 2014).

### Classification of Disordered Screen Use Behaviours

Of the selected studies, 26 examined gaming-related disorders, with 20 of them meeting the inclusion criteria for the meta-analysis. The majority used the classification Internet gaming disorder (Cai et al., 2016; Dong et al., 2015, 2017; Irak et al., 2016; Jang et al., 2021; Jeromin et al., 2016b; Li et al., 2020; Lim et al., 2016; Liu et al., 2014; Park et al., 2020; Wang et al., 2015, 2017; Wölfling et al., 2020; Wu et al., 2020; Xing et al., 2014; Yao et al., 2015; Yuan et al., 2016, 2017), whilst the remainder used Problematic Video Gaming (Collins & Freeman, 2014; Irvine et al., 2013), Internet gaming addiction (Ding et al., 2014), problematic online gaming addiction (Han et al., 2012), problematic gaming (Luijten et al., 2015), Addicted First-Person Shooter Gaming (Metcalf & Pammer, 2014), and excessive Internet gaming (Jeromin et al., 2016a; Pawlikowski & Brand, 2011). Fourteen studies examined Internet-related disorders, with 12 included in the meta-analysis. The majority used the terminology Internet addiction disorder (Choi et al., 2013, 2014; Dong et al., 2011, 2014; Shafiee-Kandjani et al., 2020; Wang & Cheng, 2020; Zhou et al., 2013, 2014, 2016), and the remainder either used problematic Internet use (Marín Vila et al., 2018; Zhou et al., 2010), Internet addiction (Kuo et al., 2018; Tekın et al., 2018), or excessive Internet use (Sun et al., 2009). Two studies examined social media addiction using either the terminology of Problematic Social Networking Sites Use (Aydın et al., 2020) or Problematic Social Network Use (Müller et al., 2021), with only the former included in the meta-analysis. Lastly, one study examined smartphone addiction (Khoury et al., 2019) and was included in the meta-analysis.

There was heterogeneity among the screeners used to assess disordered screen use. Some studies included more than one screener. The most common screeners were the Young’s Internet Addiction Test (IAT; *n* = 12), the DSM criteria (*n* = 10), the modified Diagnostic Questionnaire for Internet Addiction (YDQ; *n* = 7), and the Game Addiction Scale (GAS; *n* = 3). There were 13 screeners that were used once across the studies. There was an inconsistency in the thresholds applied to define addiction or disordered screen use. For instance, some studies implemented a cut score above 70 on the IAT to indicate addiction (Choi et al., 2013, 2014; Lim et al., 2016), whilst other studies used a score of 50 (Cai et al., 2016; Dong et al., 2015, 2017; Pawlikowski & Brand, 2011; Wang et al., 2017, 2020; Xing et al., 2014). One study implemented a 15% cut-off for the extreme scorers on the Chinese Internet Addiction Scale (CIAS) to signify addiction (Kuo et al., 2018) whilst another used scores of 67 and above as a threshold (Yao et al., 2015). Some screeners were used interchangeably to measure disordered behaviour. For example, the YDQ and CIAS were used to define both Internet addiction disorder and Internet gaming disorder (Kuo et al., 2018; Wang et al., 2015; Yao et al., 2015; Zhou et al., 2013).

### Neuropsychological Measures

There were 58 different neuropsychological tests employed with 134 unique outcome measures across all studies, with the majority examining executive functioning and attention. The most common assessment tasks were the Stroop task (*n* = 15) and the Go/No-go paradigm (*n* = 12) followed by the Stop Signal task (*n* = 5) and the WCST (*n* = 5). Approximately half of the studies included a singular neuropsychological assessment task (*n* = 21). There were 14 studies that included at least three assessments whilst the most tasks implemented in a single study were 15. In eight studies, at least one manual neuropsychological measure was used, but the majority relied solely on computerised testing.

Neuropsychological tasks and their outcome measures were sorted into cognitive domains based on the inclusion criteria for the meta-analysis (Table 3). Most tasks assessed executive functioning. There was heterogeneity in the methodology for some of the implemented neuropsychological tasks. Taking the Go/No-go task as an example, two out of the seven studies that measured the No-go error rate as an outcome did not include practice trials (Ding et al., 2014; Luijten et al., 2015) and three did not involve reward contingencies (Ding et al., 2014; Luijten et al., 2015; Sun et al., 2009). Studies used different stimuli including letters (Ding et al., 2014; Dong et al., 2010; Luijten et al., 2015), shapes (Li et al., 2020; Liu et al., 2014), and numbers (Sun et al., 2009; Zhou et al., 2014, 2016). Additionally, stimuli duration ranged from 200 (Li et al., 2020) to 1000 ms (Zhou et al., 2014, 2016) and the frequency of target trials (for No-go) ranged from 12 (Luijten et al., 2015) to 50% (Li et al., 2020). Similar variabilities were also found in the Stroop task between stimuli colour (Dong et al., 2014; Wang et al., 2015), target presentation duration (Luijten et al., 2015; Xing et al., 2014; Yuan et al., 2016), rest periods (Luijten et al., 2015; Xing et al., 2014; Yuan et al., 2016), task rewards (Dong et al., 2014), and number of trials (Dong et al., 2014; Kuo et al., 2018; Luijten et al., 2015; Wu et al., 2020; Yuan et al., 2016), with some not reporting methodologies of task administration at all (Lim et al., 2016; Tekın et al., 2018).

### Risk of Bias in Studies

Overall results are displayed in Table 2. Almost all studies had clearly defined and objective outcomes measures that were also consistently implemented (Q11, *n* = 41; although a number contained novel, *n* = 5, or experimental measures, *n* = 2), and all contained clearly defined exposure measures that were implemented consistently across study participants (Q9, *n* = 43). Most of the included studies had a clear research question or objective related to neuropsychological testing (Q1, *n* = 35), although a few focused mainly on neuroimaging and so did not have clear neuropsychological testing objectives (*n* = 8). Most studies included a clear identification of the sample (Q2, *n* = 40) that contained demographic, location, or time period recruitment descriptions. Most studies included uniform requirements for sample selection and inclusion and exclusion criteria (Q4, *n* = 36), whereas some studies only reported criteria for one group (*n* = 2), did not pre-specify criteria (*n* = 2), or did not report at all (*n* = 3). Around half of the studies had samples larger than 25 participants (Q5b, *n* = 22) and reported measuring and adjusting for potential confounding variables relevant to neuropsychological testing (Q14, *n* = 19). Areas of consistent weakness included a failure to provide sample size justification (Q5a, *n* = 4) or assess severity levels of disordered use behaviour (Q8, *n* = 8).

**Table 2:** Quality assessment of individual studies

**Study**	**Q1**	**Q2**	**Q4**	**Q5 a/b**	**Q8**	**Q9**	**Q11**	**Q14**
Aydin et al. (2020)	Y	Y	Y	N/Y	N	Y	Y	Y
Cai et al. (2016)	Y	Y	N^c^	N/Y	N	Y	Y	N^g^
Choi et al. (2013)	Y	Y	Y	N/N	N	Y	Y	N
Choi et al. (2014)	Y	Y	Y	N/N	N	Y	Y	Y
Collins and Freeman (2014)	Y	Y	Y	N/N	Y	Y	Y^e^	N
Ding et al. (2014)	Y	Y	Y	N/N	N	Y	Y	N^g^
Dong et al. (2010)	N^a^	N^b^	Y	N/N	N	Y	Y	N^g^
Dong et al. (2014)	Y	Y	N^d^	N/N	N	Y	Y	N
Dong et al. (2015)	N^a^	Y	N^d^	Y/N	N	Y	Y	N^g^
Dong et al. (2017)	Y	Y	Y	N/N	Y	Y	Y^e^	N^g^
Han et al. (2012)	N^a^	Y	N^c^	N/N	Y	Y	Y	Y
Irak et al. (2016)	Y	N^b^	Y	N/Y	Y	Y	Y^e^	N
Irvine et al. (2013)	Y	Y	Y	N/N	N	Y	Y^e^	Y
Jang et al. (2021)	Y	Y	Y	N/Y	Y	Y	Y	Y
Jeromin et al. (2016a)	Y	Y	Y	Y/N^c^	N	Y	Y^f^	N
Jeromin et al. (2016b)	Y	Y	Y	N/Y	N	Y	Y^f^	N
Khoury et al. (2019)	Y	Y	Y	Y/Y	N	Y	Y	Y
Kuo et al. (2018)	Y	Y	Y	N/Y	N	Y	Y	Y
Li et al. (2020)	Y	Y	Y	N/Y	N	Y	Y^e^	Y
Lim et al. (2016)	Y	Y	Y	N/Y	N	Y	Y	Y
Liu et al. (2014)	N^a^	Y	Y	N/N	N	Y	Y	N
Luijten et al. (2015)	Y	N^b^	Y	N/N	Y	Y	Y	N
Marin Vila et al. (2018)	Y	Y	Y	Y/Y	N	Y	Y	Y
Metcalf et al. (2014)	Y	Y	Y	N/ N^c^	Y	Y	Y	Y
Muller et al. (2021)	Y	Y	Y	N/Y	N	Y	Y	Y
Pawlikowski et al. (2011)	Y	Y	Y	N/N	N	Y	Y	Y
Park et al. (2020)	Y	Y	Y	N/Y	N	Y	Y	Y
Shafiee-Kandjani et al. (2020)	Y	Y	Y	N/Y	N	Y	Y	N
Sun et al. (2009)	Y	Y	Y	N/Y	N	Y	Y	Y
Tekin et al. (2018)	Y	Y	Y	N/Y	N	Y	Y	N
Wang et al. (2015)	Y	Y	NR	N/Y	N	Y	Y	N
Wang et al. (2017)	N^a^	Y	Y	N/N	N	Y	Y	N
Wang et al. (2020)	Y	Y	Y	N/N	N	Y	Y	N
Wolfing et al. (2020)	Y	Y	Y	N/Y	Y	Y	Y	Y
Wu et al. (2020)	Y	Y	Y	N/Y	N	Y	Y	Y
Xing et al. (2014)	Y	Y	Y	N/N	N	Y	Y	N
Yao et al. (2015)	Y	Y	Y	N/Y	N	Y	Y	Y
Yuan et al. (2016)	N^a^	Y	Y	N/Y	N	Y	Y	N
Yuan et al. (2017)	N^a^	Y	Y	N/Y	N	Y	Y	Y
Zhou et al. (2010)	N^a^	Y	Y	N/Y	N	Y	Y	N
Zhou et al. (2013)	Y	Y	Y	N/N	N	Y	Y	N
Zhou et al. (2014)	Y	Y	N	N/N	N	Y	Y	N
Zhou et al. (2016)	Y	Y	N	N/N	N	Y	Y	N

Risk of bias in individual studies. *Q1* Was the research question or objective in this paper clearly stated? *Q2* Was the study population clearly specified and defined using demographics, location, and time period? *Q4* Were all the subjects selected or recruited from the same or similar populations (including the same time period)? Were inclusion and exclusion criteria for being in the study pre-specified and applied uniformly to all participants? *Q5a* Was a sample size justification, power description, or variance and effect estimates provided? *Q5b* Was sample size ≥ 25 per group? *Q8* Did the study examine different levels of the exposure as related to the outcome? *Q9* Were the exposure measures clearly defined, valid, reliable, and implemented consistently across all study participants? *Q11* Were the outcome measures clearly defined, valid, reliable, and implemented consistently across all study participants? *Q14* Were key potential confounding variables measured and adjusted statistically for their impact on the relationship between exposure(s) and outcome(s)? *a* focus was neuroimaging. *b* did not include location or time period. *c* for one group. *d* not pre-specified. *e* novel measure. *f* experimental analysis. *g* only for neuroimaging. *NR* not reported. *Y* yes. *N* no

### Synthesis of Results

**Fig. 2:**
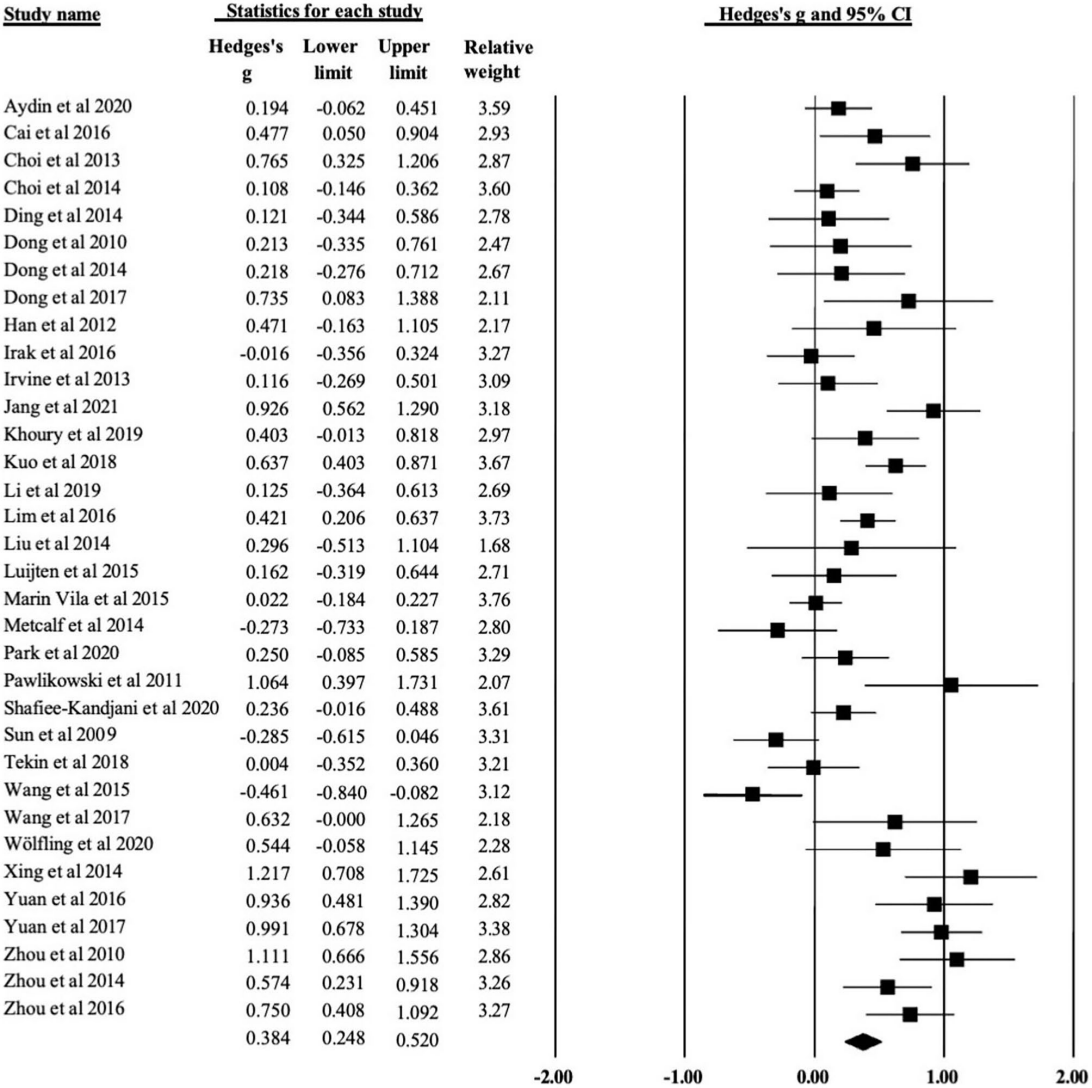
Forest plot of individual study effect sizes among studies of cognitive performance between disordered use behaviour and controls showing Hedges’ *g* based on a random-effects model* g*, relative weightings, and 95% confidence intervals reflected by error bars

Figure 2 displays the range of study effects comparing performance on cognitive tasks between participants with screen disorder to healthy controls. The analysis included 34 cross-sectional observational studies across 1076 participants with disordered screen use behaviour and 1338 healthy controls. Across all studies and tests of cognition, those with disordered screen use behaviour had significantly lower cognitive performance scores compared to controls, resulting in a mid-range small Hedges’ *g* effect size,* g* = .38, 95% CI (.25, .52), *p* < .001. There was evidence of significant considerable heterogeneity between the studies (*Q* = 145.52, *p* < .001, *I*^2^ = 77.32, *τ*^2^ = .34) suggesting the need for further investigation of this heterogeneity through subgroup analyses.

### Cognitive Domain Analysis

**Fig. 3:**
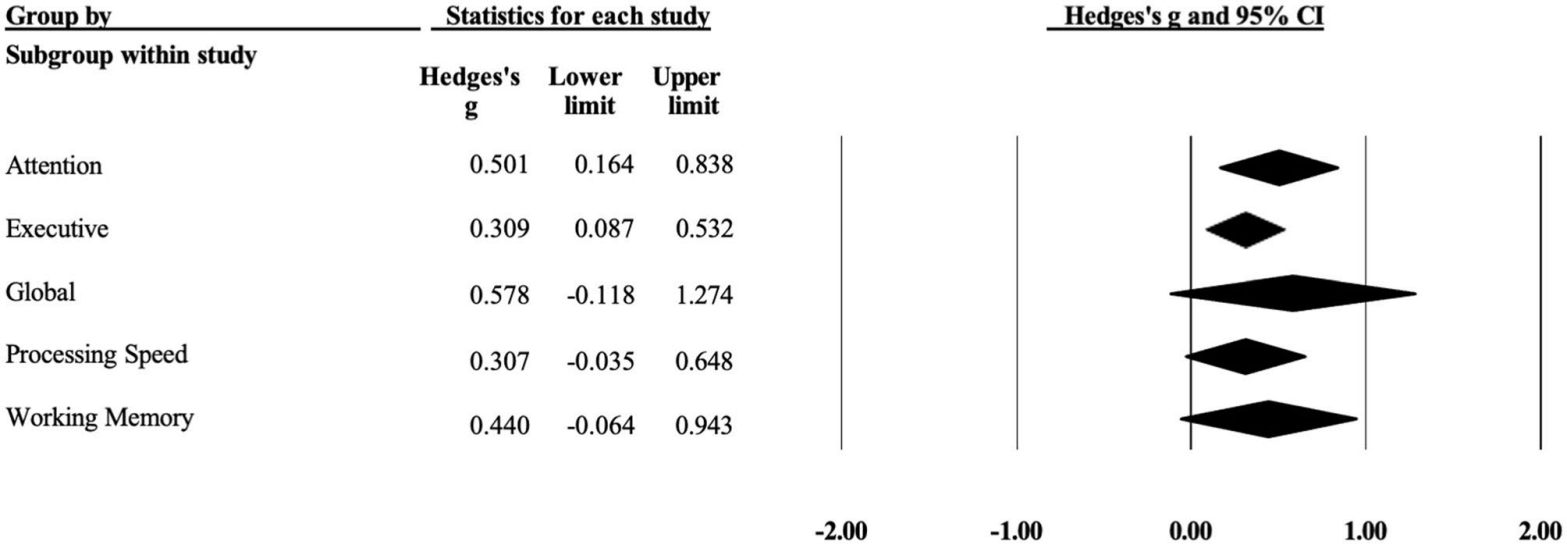
Forest plot for the meta-analysis of overall cognitive performance of all included studies grouped by cognitive domain, showing Hedges’ *g* based on a random-effects model and 95% confidence intervals

A subgroup analysis was conducted to examine the differences between disordered screen use and control samples by the different cognitive domains (Fig. 3). Executive functioning was the most assessed domain with 32 studies overall, followed by 14 studies that assessed attention, 13 studies that measured processing speed, six studies that measured working memory, and three studies that assessed global functioning. Relative to healthy controls, individuals with disordered use showed significant moderate impairment in the domain of attention (*g* = .50, 95% CI [.16, .84], *p* = .004, *I*^2^ = 71.84, *τ*^*2*^ = .46) and significant small impairment in executive functioning (*g* = .31, 95% CI [.087, .53], *p* = .006, *I*^2^ = 87.64, *τ*^*2*^ = .73). There were no significant differences between individuals with disordered use and controls in the domains of processing speed (*g* = .31, 95% CI [− .037, .65], *p* = .080, *I*^2^ = 69.96, *τ*^*2*^ = .36), working memory (*g* = .44, 95% CI [− .064, .94], *p* = .089, *I*^2^ = 35.00, *τ*^*2*^ = .19), or global functioning (*g* = .58, 95% CI [− .12, 1.28], *p* = .11, *I*^2^ = 74.22, *τ*^*2*^ = .39). To determine whether there was any difference in the effect sizes between the cognitive domains, a mixed effects analysis revealed there was no significant difference (*Q* = 1.41, *p* = .84).

### Test Level Analysis

To examine which test performances were most impacted for the disordered screen use samples relative to controls, we ran an analysis across every individual test (Table 3). Out of 134 unique neuropsychological outcome measures, 32 were computable in the quantitative analysis according to the inclusion criteria. For example, several tasks were included only once in all studies, such as the Cups Task, Cambridge Gambling Task, and flanker compatibility task, and some outcome measures were either only used once or included a non-traditional, experimental outcome. For tasks assessing executive functioning, there were significantly reduced performances for individuals with disordered screen use on all measures on the WCST, as well as reduced accuracy scores for incongruent trails on the Stroop task, Delay Discounting task, and proportion of successful stops on the Stop Signal task. Interestingly, the disordered screen use sample had significantly quicker reaction times than controls for No-go trials on the Go/No-go task requiring rapid impulse control. The most diminished performance for individuals with disordered screen use was on the go trial on the Go/No-go task assessing attention with a significant large effect size of 1.28. It should be noted this was significantly influenced by Zhou et al. (2010) with a Hedges’ *g* effect size of 4.0 for this task. There was also a significant medium/large reduction in performances on the forward recall Digit Span task and go trials on the Stop Signal task. Interestingly, there were no significant differences on the Go/No-go and Stop Signal tasks that measured reaction times as an outcome. Relatedly, there were no significant differences between disordered screen use and control samples on tests of processing speed. In the working memory domain, backward recall and the composite index of the Digit Span task were significantly reduced. There were no significant differences on the Spatial Span task. Lastly, within the global domain of cognition, performances were reduced for individuals with disordered screen use as measured by the Full-Scale IQ index on the WAIS.

**Table 3:** Test level analysis

Cognitive domain	Neuropsychological test	*n* studies	Hedges’ *g*	Standard error	Lower	Upper	*p*-value
Executive functioning	Delay Discounting task	3	.59	.17	.26	.92	.000
	Game of Dice task	2	.58	.44	− .29	1.45	.194
	Go/No-go no ER	9	.01	.47	− .91	.93	.977
	Go/No-go no RT	2	− .48	.19	− .86	− .10	.014
	Intra-Extra Dimensional Set Shift	2	.18	.17	− .16	.52	.293
	Iowa Gambling Test	3	.39	.25	− .10	.89	.121
	Stop Signal task POSS	4	.52	.25	.02	1.01	.040
	Stop Signal task stop RT	3	.18	.15	− .11	.46	.229
	Stroop incongruent ER	6	1.20	.39	.43	1.96	.002
	Stroop incongruent RT	11	.38	.21	− .02	.78	.063
	Stroop response delay	5	.44	.38	− .31	1.19	.249
	Trails Making test B	4	.058	.15	− .24	.36	.701
	Verbal Fluency task	3	.15	.15	− .14	.44	.300
	WCST CA	3	.29	.11	.08	.51	.008
	WCST CLR	3	.94	.16	.63	1.26	.000
	WCST FMS	2	.96	.22	.53	1.39	.000
	WCST PE	5	.80	.33	.14	1.45	.017
	WCST TE	3	.75	.24	.27	1.22	.002
Attention	Digit Span forwards	4	.70	.35	.57	1.95	.000
	Go/No-go go ER	7	1.28	.69	1.71	4.40	.000
	Go/No-go go RT	9	.021	.23	− .44	.48	.927
	Stop Signal task go errors	2	.70	.22	.28	1.12	.001
	Stop Signal task go RT	2	− .30	.30	− .90	.29	.316
Processing speed	Stroop congruent ER	3	− .05	.15	− .34	.24	.747
	Stroop congruent RT	3	.34	.19	− .04	.73	.078
	Trails Making test A	4	.16	.13	− .09	.41	.200
	Symbol Digit Modalities Test	2	.03	.10	− .16	.22	.740
Working memory	Digit Span backwards	4	.56	.13	.29	.82	.000
	Digit Span composite	3	.42	.16	.10	.74	.011
	Spatial Span length	2	− .03	.18	− .39	.32	.856
	Spatial Span total errors	2	.02	.17	− .32	.36	.918
Global	WAIS (FSIQ)	3	.59	.26	.08	1.11	.023

*CA* categories achieved, *CLR* conceptual level responses, *FMS* failure to maintain set, *FSIQ* full-scale IQ, *POSS* proportion of successful stops, *WAIS* Wechsler Adult Intelligence Scale, *PE* preservative errors, *RT* reaction time, *TE* total errors

### Testing Format Analysis

A subgroup analysis examined the difference between the effect sizes for computerised and manual testing for disordered screen use compared to controls. There was a small significant Hedges’ g effect size (*g* = .37, 95% CI [.22, .52], *p* < .001) for computerised testing and a small significant effect size for manual testing (*g* = .35, 95% CI [.080, .63], *p* = .013). However, the two formats of testing did not differ significantly from each other (*Q* = .002, *p* = .90). Overall, the 29 studies that reported the use of computerised testing significant considerable heterogeneity was found (*Q* = 121.30, *I*^2^ = 76.92, *τ*^*2*^ = .38, *p* < .001). The seven studies that reported manual testing were likewise considered heterogeneous (*Q* = 30.68, *I*^2^ = 80.44, *τ*^*2*^ = .27, *p* < .001).

### Addiction Type Analysis

A subgroup analysis was run to examine whether disordered use classification moderated cognitive outcomes for individuals with disordered screen use behaviours compared to controls. The singular studies that examined social media addiction (Aydın et al., 2020) and smartphone addiction (Khoury et al., 2019) were excluded from the analysis given a needed minimum of two studies for quantitative analysis. There was significant heterogeneity found for the 19 studies that examined gaming addiction (*Q* = 86.89, *I*^2^ = 79.28, *τ*^*2*^ = .41, *p* < .001). The 13 studies that examined Internet addiction were likewise considered heterogeneous (*Q* = 59.35, *I*^2^ = 79.78, *τ*^*2*^ = .33, *p* < .001). We found a medium significant Hedges’ *g* effect size (*g* = .40, 95% CI [.21, .60], *p* < .001) for gaming addiction and a medium significant Hedges’ *g* effect size (*g* = .36, 95% CI [.14, .59], *p* = .002) for Internet-related disordered behaviour. The two types of disordered use classifications did not differ significantly (*Q* = .065, *p* = . 79).

### Exploratory Analysis

Given the ongoing debate regarding the distinction between disordered social media use and other forms of disordered screen use (see Weinstein, 2022), we conducted an exploratory analysis to investigate whether the pattern of observed outcomes would noticeably differ if we excluded social media from the analysis. After excluding Aydın et al. (2020), we found an incremental change in overall effect size of* g* = .39, 95% CI (.25, .53), *p* < .001, across 33 studies with evidence of significant considerable heterogeneity between studies (*Q* = 144.17, *p* < .001, *I*^2^ = 77.80, *τ*^2^ = .35). The cognitive domain analysis showed minor changes in executive functioning (*g* = .31, 95% CI [.082, .55], *p* = .008, *I*^2^ = 88.01, *τ*^*2*^ = .79) with no significant difference between domains (*Q* = 1.29, *p* = .86). The test level analysis revealed a change on WCST CA (*g* = .28, 95% CI [− .13, .68], *p* = . 184), which was no longer statistically significant, and a slight increase in scores on WCST PE (*g* = 1.00, 95% CI [.41, 1.59], *p* = .001). Lastly, the testing format analysis revealed an effect size of *g* = .38, 95% CI (.22, .54), *p* < .001, for computerised testing (*Q* = 120.07, *I*^2^ = 77.51, *τ*^*2*^ = .40, *p* < .001) with no significant difference between formats.

### Reporting Biases

**Fig. 4:**
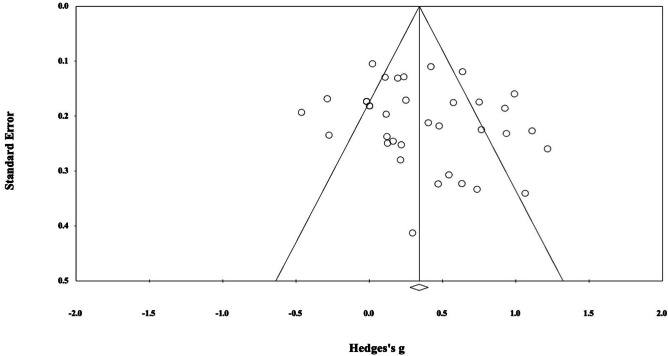
Funnel plot of all included studies’ effect sizes (Hedges’ *g*)

Risk of bias across studies was conducted. First, visual inspection of the funnel plot (Fig. 4) indicated that the distribution of studies of overall cognitive functioning is mostly symmetrical around the estimated effect size, although the studies were more clustered on the left of the effect. There was a single outlier; however, this study had the smallest sample size (*n* = 11) and had the smallest weighting on the overall results (Liu et al., 2014). Indeed, a leave-one-out analysis confirmed that when this study was removed, it had little impact on the overall effect which was still significant and small, *g* = .37, 95% CI (.23, .51). The Egger’s test for plot symmetry was not significant (*Egger’s intercept* = 1.34, *p* = .22), suggesting that publication bias did not significantly impact validity. Based on Duval and Tweedie’s trim and fill analysis, no studies are required to be trimmed from the right side, and one study should be trimmed from the left side, leading to an adjusted significant effect size of *g* = .34, 95% CI (.28, .40), indicating that bias was not detected. According to the classic fail-safe N method, there would need to be 1056 non-significant studies to produce a null effect and for the obtained effect size of *g* = .38 to be overturned (Zakzanis, 2001). Based on our observation of 34 studies, this number of unpublished studies has a very low probability. Risk of bias assessment was conducted for all subgroup analyses and the exploratory analysis with no significant results from Egger’s test suggestive of no publication bias. Funnel plots and other analyses can be found through https://osf.io/upeha/.

## Discussion

In the current systematic review and meta-analysis, we sought to synthesise and quantitatively assess the magnitude of neuropsychological deficits from disordered screen use behaviours. In particular, this was undertaken to resolve apparent inconsistencies in the neuropsychological literature concerning the cognitive impacts of disordered screen use behaviours. Indeed, with an increasing trend in problematic screen use prevalence (Pan et al., 2020), understanding the exact extent of cognitive consequences remains a vital concern. For this purpose, we identified cross-sectional studies that compared performance on objective neuropsychological tasks between disordered screen use behaviour samples and healthy controls. We explored the heterogeneity across diagnoses and neuropsychological testing, as well as appraised the quality of studies conducted. In our quantitative examination, we investigated the differences in cognitive performance as a function of cognitive domain, disordered use classification, test type, and test format. We found that individuals with disordered screen use behaviours had significantly lower cognitive performances with an effect size of .38, with attention showing the greatest reductions followed by executive functioning. This reduction was not moderated by either the classification of disordered screen use into gaming or Internet behaviours or by the format of the tests. Although almost all studies fulfilled quality requirements, these results may have been impacted by a consistent failure to provide sample size justifications and assess the severity of disordered screen use behaviours. This extended the existing literature by including a broad spectrum of cognitive abilities and neuropsychological assessment tasks as possible across all disordered screen-related behaviours, screen modalities, and ages.

### Overall Cognitive Performance

The review identified 43 cross-sectional studies and 34 were eligible for the meta-analysis. Firstly, we found that most of the included studies were of young Asian males, consistent with higher prevalence rates in Asian countries (Naskar et al., 2016) and the disproportionate prevalence of disordered screen use behaviours among younger males (Wittek et al., 2016). We found that study effect sizes varied widely in cognitive performance, from a *g* = − .46 showing better performance compared to controls to a *g* = 1.22 indicating worse performance compared to controls, a likely artefact of the variability in the neuropsychological literature. With an estimated overall study effect size score of *g* = .38, we revealed an overall reduction in cognitive performance for individuals with disordered screen use behaviours that is on the higher end of the small effect size range using historical cut-offs.

In comparing the extent of cognitive performance, the measured effect size of .38 indicates a reduction of almost half of a standard deviation compared to controls (for comparing effect size with standard deviation, see Abramovitch et al., 2013). Based on Funder and Ozer’s (2019) review of effect sizes in psychology, the estimated Hedges’ *g* effect size corresponds to a Pearson’s *r* score of .18, which by their newer criteria would indicate an effect with likely explanatory and practical relevance, even in the short term. In other words, even a small effect can compound critically across time, especially in the context of childhood education. As an example, research has shown that even minor cognitive reductions at an early age, like those caused by mild traumatic brain injury, can lead to progressively increasing lags in academic performance and further “widening of the gap” in comparison to peers (Babikian et al., 2011; Maillard-Wermelinger et al., 2010). Therefore, unless remediated, even minor reductions in cognitive performance can gradually lead to more profound impairments across time.

Whilst we have found a reduction in cognitive performance, the extent of that reduction remains unclear. Abramovitch and Cooperman (2015) highlight that when interpreting effect sizes, underperformance on test scores does not necessarily imply clinically significant functional impairment. Based on Taylor and Heaton’s (2001) recommendation, a standard deviation of 1.0 is typically a useful diagnostic criterion for capturing neuropsychological impairment with specificity and sensitivity. However, given that only four studies had an effect size over one, the extent to which individuals with disordered screen use have clinically significant impairments without ecologically valid tests of impairment remains unclear. For measuring the extent and nature of cognitive reduction as reflected by clinically significant functional impairments, it would be beneficial for future studies to conduct ecologically valid assessments in the context of everyday functioning in academic, professional, and other real-world settings (see Spooner & Pachana, 2006). Nonetheless, given the critical age in which this reduction in cognitive performance is seen to take place, it seems important that some form of remediation be administrated to ensure that these reductions in cognition do not compound over time.

### Cognitive Domains

We found that the most profound deficits for individuals with disordered screen use behaviours were found in the domain of attention. From a cognitive standpoint, attention is considered foundational to other aspects of thinking as it is the cognitive bottleneck for both processing incoming information and deploying attentional resources outwards (Luria, 1980; Mapou, 1995). Indeed, it is common for neuropsychologists to examine arousal and attention first during assessment, as how well a person can pay attention determines how much information they can process, attend to, or commit to memory, and deficits in these areas are likely to impact all other cognitive functions (Mapou, 1995). Given that attention was most impaired, we might expect to see broader global impairments in cognitive functioning. Indeed, we found that there was no significant difference between cognitive domains suggesting a trend of global impairment. It is also important to consider that cognitive domains are not isolated and separate constructs but can be highly correlated and dynamically related. Therefore, decrements measured in one domain may be interdependent to reductions in other domains. However, the extent to which attention was producing more broad level impairment in cognition remains unclear. In addition, whilst we have grouped tasks into a broad domain of attention, it is necessary to examine how and whether disordered screen use may impact the various subtypes of attention differently (see Salo et al., 2017; van Zomeren & Brouwer, 1992).

One possibility is that whilst there may be deficits in a global definition of attention, there may be increases in selective attention or divided attention. For instance, video game players, characterised by at least 7 h of gaming a week across 2 years, will either outperform or demonstrate no difference from non-gamers on some tasks of attention (Boot et al., 2008). Gaming has also been linked to increases in correctly filtering out irrelevant items (divided attention) and recovering from attention shifts (Moisala et al., 2017). Indeed, for tasks that require singular focus and successful inhibition of automatic impulses, gamers tend to perform worse than non-gamers, whereas for tasks that require filtering out stimuli and shifting attention, gamers tend to outperform non-gamers (DeRosier & Thomas, 2018). However, these possible variabilities within cognitive domains may have been overlooked in this review which took a broad analysis point of view. Future studies with comprehensive neuropsychological batteries are needed to determine whether decrements in attention result in more global cognitive changes or whether the less frequently studied subdomains and domains (such as language and memory) will also follow the observed pattern of impairment. Additionally, given the known interdependencies and interactions between cognitive domains (Engle et al., 1999; Unsworth & Engle, 2007; Unsworth et al., 2009), investigating the impacts of disordered screen use from a global cognition perspective using advanced techniques such as network analysis that account for these interdependencies can offer a more comprehensive understanding of the cognitive impacts of disordered screen use behaviours (Kellermann et al., 2016).

### Individual Test Type

In order to identify which tests led to the greatest underperformances, we analysed individual neuropsychological task performances comparing disordered screen use and control samples. For disordered screen users, accuracy scores on the go condition of the Go/No-go task showed the greatest underperformance with a significant large effect size, followed by the forward condition on the Digit Span task. From those studies, one included statistical adjustment for potential confounding variables and two examined levels of severity, thereby limiting inferences about causality. Both tasks share a similarity in that successfully responding to go trials as well as listening to and repeating a sequence of digits requires vigilance, concentration, and sustained attention (Hale et al., 2016; O’Connell et al., 2009). The act of maintaining one’s attention over time requires the dual abilities to both allocate attentional resources and reorient attention as it strays (van Zomeren & Brouwer, 1992). The dynamic, captivating, and visually stimulating features characteristic of screen-based media and technologies may challenge the capacity to both focus and reorient attention towards information that are more mundane and a lot less stimulating and rapidly changing, such as the Go/No-go and Digit Span tasks. Indeed, even a single night of fast-paced, action binge video gaming can result in reduced performances on a sustained attention task (Trisolini et al., 2018). Interestingly, we found that disordered screen users had enhanced reaction times on No-go trials for the Go/No-go task compared to controls. In comparison to go trials, successfully responding to No-go trials requires the rapid inhibition of automatic responding (O’Connell et al., 2009). It is possible that the same elements of screen-based media and technologies that can disadvantage attention may be advantageous for rapid response inhibition (see Dye et al., 2009). Regarding overall inhibitory control, however, it was found that disordered screen users had significantly reduced performances on the WCST and for incongruent trials on the Stroop task compared to controls. In sum, the most reduced performances for disordered screen users were on neuropsychological tasks that required sustained attention, although similar underperformances were also evident on specific tasks of executive functioning, working memory, and global functioning.

### Neuropsychological Considerations

Among the included studies, we found that the methodologies used for the cognitive tasks were highly variable in terms of stimulus durations, reward contingencies, target stimuli, and target frequencies. Standardisation and consistent procedures in cognitive testing are crucial, largely due to the emphasis on comparing an individual test performance against a normative standard, but also to ensure scientific rigour and inter-rater and test–retest reliability (Russell et al., 2005). Using the Go/No-go task as an example, as in this study, Wessel (2018) found that the administration of the task including the frequency of targets and the duration of stimuli tended to vary widely across studies. Critically, electroencephalography (EEG) event-related potential (ERP) measurements revealed that even seemingly minor differences in task administration engaged separate neural processes, thereby emphasising the need to conduct consistent testing. Whilst we have found significant underperformances on the Go/No-go task, the variability in task administration is a critical consideration when interpreting these results. Given the degree of heterogeneity in cognitive task administration, we have found that there is a clear need to administer consistent, standardised, and previously validated assessments rather than modifying or creating novel assessment tasks.

Moreover, nearly half of the included studies used a singular neuropsychological task to assess a cognitive domain. There are several neuropsychological implications to consider when interpreting test results from single-test studies or when there is an over-reliance on a single test across studies. Lezak et al. (2012) argue that use of a single test to identify a disorder or impairment, both within studies and across studies, can lead to higher rates of impairment misclassification. For one, the absence of a positive finding does not automatically preclude the possibility of a present impairment in the same way that the presence of a negative finding (on a single test) does not automatically presume cognitive impairment. For example, a reduced score on the incongruent condition of the Stroop task does not automatically imply, as some studies put it “impaired cognitive control” (Cai et al., 2016, p. 16) or “cognitive control deficits” (Yuan et al., 2017, p. 5). Rather, in the case of making an inference about the broad domain of executive functioning, an evaluation must be made based on the pattern of test scores and across different tests of executive functioning (Lezak et al., 2012). Otherwise, a reduced score tells us something about the specific key process involved in a given test and less about the domain of interest. Additionally, drawing inferences about cognitive impairment from the findings of a single test is heavily dependent on the psychometric integrity and sensitivity of the test in question.

As discussed above, heterogeneity in test administration can challenge the psychometric integrity of a test, an estimate based on standardisation. Studies would benefit from including a test of batteries that are standardised, sensitive, and specific to the nature of impairment that will more suitably allow for the possibility of detecting patterns of deficits within and across cognitive domains in an addicted population.

### Modality of Testing

It has been previously demonstrated that individuals with disordered screen use behaviours exhibit an attentional bias towards computer or screen-related stimuli (Heuer et al., 2021; Kim et al., 2018). Awareness of such an attentional bias could be crucial when choosing appropriate neuropsychological assessments to measure cognition. Indeed, the “best practice” guidelines in neuropsychological assessment require that scores reflect a participant’s best performance and that tests are acceptable to assess the relevant functions (Bush, 2009). If extraneous variables are present, such as suboptimal effort or some source of distraction, then these should be accounted for either in mention or through psychometric scoring (e.g., adjusted according to level of attention). To our knowledge, whether the attentional bias towards computer-related or screen-related stimuli impacts performance on computerised neuropsychological testing has hitherto neither been questioned nor investigated.

Despite the above concerns regarding appropriateness of testing, we found that only eight out of 43 studies included at least one manual neuropsychological measure whilst the rest relied solely on computerised testing. As part of our analysis, we also conducted a subgroup analysis to determine whether the two types of cognitive testing, manual and computerised, moderated cognitive performance. Computerised tasks had a slightly larger effect size than manual testing; however, there was no significant difference in cognitive performance between the two formats of administration. In other words, the format of administration did not produce any advantage or disadvantage on cognitive performance or for detecting cognitive impairment in disordered screen use. Although we found that cognitive performance between groups did not significantly differ as a function of testing format, there was an overwhelming majority of computerised studies. More studies utilising different formats of neuropsychological testing, such as paper-and-pencil, computerised, and virtual-reality, would be useful in examining the contribution of format to cognitive performance in a disordered screen use population.

### Classification of Disordered Screen Use Behaviours

This review found that although there were overall strengths in defining, describing, and using validated measures, there was a high degree of variability in the methods employed to describe and classify disordered screen use behaviours. The two most common classifications were IGD and IAD. However, 16 studies included diagnostic classifications that were used twice or less across all studies. The classification measures and cut-offs were also applied inconsistently. As mentioned above, the YDQ measurement scale was used to define both IAD and IGD. This is consistent with a previous systematic review which found a considerable degree of variability in implemented diagnostic measures for classifying gaming disorders as well as a tendency for studies to adapt or create new measures rather than adopt previously validated ones (King et al., 2020).

Although the degree of inconsistency and variability is unsurprising given the relatively recent exploration of screen-related disorders as a field of study, there is a clear imperative for consistency in disordered screen use measurement that has direct implications. As an example, a meta-analysis found that studies utilising the IAT or CIAS estimated higher prevalence rates of gaming disorder than studies that employed the YDQ measurement scale (Li et al., 2018). To address the varying quality of screener measures, Koronczai et al. (2011) suggested that disordered screen use measurement tools be brief, comprehensive, reliable, valid for all ages, cultures, and data collection methods, as well as clinically validated to be able to broadly apply across countries, screen modalities, and variables of interest.

Our analysis study aimed to examine the magnitude of cognitive impairment as a function of disordered screen use classification. Our findings showed that, although the estimated effect size for gaming-related disordered behaviour was slightly larger than for studies including Internet-related behaviours, this difference was not significant. In other words, from a neuropsychological standpoint, the classification of disordered screen use behaviours according to the predominant modality of usage (Internet or gaming) did not moderate the magnitude of cognitive impairment. However, as only one study examined disordered social media use behaviours and one study examined disordered smartphone use behaviours, there were not enough studies in each category to estimate an effect size for either. Nevertheless, excluding social media in an exploratory analysis revealed only marginal changes across the overall and relevant subgroup results. Interestingly, the results indicated slightly poorer performance in individuals with disordered screen use compared to controls when social media was excluded. This suggests that the cognitive effects of problematic social media use may not be as severe as those associated with other forms of screen use, which is consistent with other findings (see Weinstein, 2022). However, since only minor changes were observed, this finding lends some support to grouping social media with other forms of problematic screen use when assessing their impact on cognition. Still, due to the inclusion of only one social media study, we could not determine the significance of the differences between classifications. Further studies are needed that assess other forms of problematic screen use, including social media, before such a conclusion can be made.

Further, our review identified only eight studies that presented severity classifications for disordered screen use behaviour. This limits the extent to which the relationship of cognitive performance across a spectrum of severity behaviours can be investigated. A previous systematic review on Internet gaming disorder in children and adolescents recommended that researchers make a distinction between levels of engagement with gaming (Paulus et al., 2018). As the authors make clear, any psychosocial and academic consequences may vary significantly based on levels of engagement, with even high levels of engagement resulting in some positive effects. For these reasons, more studies that examine a range of screen modalities across a continuum of severity, especially in terms of causally linking severity of disordered screen use behaviours to cognitive impacts, are needed to establish a relationship.

### Limitations

There are several important limitations to consider. First, although we have shown that attention and executive functioning are impaired in disordered screen use, we were not able to comprehensively cover all cognitive domains in this meta-analysis (e.g., memory, visuospatial ability, or language) nor were we able to confidently examine subcomponents of cognitive domains (e.g., selective attention, divided attention, nonverbal reasoning, decision-making, or impulse inhibition) owing to the limited number of studies that examined those domains. Additionally, whilst we have followed clinical guidelines in sorting the tests under their relevant domains, there is no definitive consensus about which cognitive domains a test truly measures. There is also considerable overlap and correlation between cognitive domains, which can make it difficult to categorise tests definitively. Second, we did not search for unpublished studies concerning cognitive impacts of disordered screen use that may exist. Third, it is important to consider that the overwhelming majority of included studies were of an Asian, young, and male demographic, thereby limiting the global generalisability of these results particularly in older, non-male, and Western populations. For one, the prevalence and severity of disordered screen use behaviours are known to be higher in Asian countries compared to Western countries (Naskar et al., 2016). Given that the diagnostic criteria for IGD or IAD do not establish severity of symptoms beyond the cut-offs, cognitive impacts might be more extreme in Asian populations. Moreover, culture and gender can impact the expression and distress resulting from disordered screen use behaviours, so a broad range of cultures and genders is essential for generalisation (Andreetta et al., 2020; Kuss, 2013). Another limitation stems from the inherent constraints of cross-sectional studies, which limits a more thorough understanding of the contributions of moderating variables. For instance, it remains uncertain whether factors such as anxiety or depression, known to have a high comorbidity with disordered screen use behaviour (with rates as high as 92% and 89% respectively, see González-Bueso et al., 2018), precipitate increased screen usage or result from it. Lastly, we found that very few studies included a sample size justification (although almost half had a sample size greater than 25), assessed the severity of disordered screen use behaviours, or statistically adjusted for potential confounding variables. Along with the narrow range of the measured population and this review’s focus on cross-sectional observational studies, this limits a greater understanding of causality and the contribution of other variables.

### Future Recommendations

Future studies should consider the following recommendations. In order to identify and evaluate disordered screen use, researchers should use consistent and validated methods rather than modifying or adopting novel screening measures and cognitive tasks. Second, research on neuropsychological impacts would benefit from a battery of cognitive tests that measure the range of cognitive functioning across and within cognitive domains rather than relying on and interpreting results based on a single test. Third, assessing the severity of disordered screen use behaviours will provide insight into and possibly establish a relationship between cognitive deficits and symptom severity. Fourth, to establish a causal or prospective relationship between disordered screen use behaviours and cognitive impacts, future investigations should consider adopting experimental and longitudinal designs. Fifth, ecologically valid assessments of cognitive functioning should be incorporated to determine the severity of impairments experienced in daily life. Sixth, although disordered screen use is more prevalent in some demographics, little is known about its cognitive impacts on older, non-male, and Western populations. It would be beneficial to investigate these underexplored populations in future research.

### Conclusion

In summary, the results of this systematic review and meta-analysis suggest that disordered screen use can negatively impact cognitive abilities. Attention is the most affected cognitive domain, followed by executive functioning, but further research is needed to determine the magnitude of deficits in other lesser-studied domains. Neither disordered screen use classification nor testing format influenced the extent of cognitive deficits from a neuropsychological perspective. However, given the limited number of studies, more research that incorporates broader disordered screen use behaviours, including social media and smartphones, and includes comprehensive manual cognitive assessments are required. With increased reliance on technology, it has never been more important to assess the impact of too much use of screens on cognitive functioning and overall wellbeing. This will enable the development of targeted remediation and treatment plans as well as inform designer decisions regarding development of technological platforms and devices with cognitive impacts in mind.

## Data Availability

All of the reviewed studies are publicly available. Data, figures, and results from this study are available through https://osf.io/upeha/.
